# Changes in functional capacity, flexibility and depressive symptoms in women with breast cancer submitted to adjuvant radiotherapy: randomized controlled clinical trial with mat pilates

**DOI:** 10.1007/s00520-026-11448-6

**Published:** 2026-11-14

**Authors:** Daniele Medeiros Torres, Kelly de Menezes Fireman, Erica Alves Nogueira Fabro, Luiz Claudio Santos Thuler, Anke Bergmann, Sabrina da Silva Santos

**Affiliations:** 1 National Institute of Cancer - INCA, Hospital Do Câncer III, Visconde de Santa Isabel, Rio de Janeiro, RJ 274 Brazil; 2 National Public Health School (ENSP, Escola Nacional de Saúde Pública), Oswaldo Cruz Foundation (FIOCRUZ), Rio de Janeiro, RJ Brazil; 3 Graduate Program in Applied Health Sciences, Universidade de Vassouras, Vassouras, Rio de Janeiro, Brazil

**Keywords:** Breast Neoplasms, Radiotherapy, Exercise, Pilates Based Exercises

## Abstract

**Purpose:**

To evaluate the impact of Mat Pilates on functional capacity, flexibility, and depressive symptoms in women diagnosed with breast cancer who received adjuvant radiotherapy.

**Methods:**

In this randomized clinical trial, 164 women with breast cancer (stages 0–III), aged 18 years or older, were randomized, and 156 were included in the analyses (79 in the Mat Pilates group and 77 in the control group). Handgrip strength, functional exercise capacity assessed by the 2-Minute Step Test (2MST), flexibility, and depressive symptoms were evaluated at baseline, at the end of radiotherapy (RT), and at 30 days, 3 months, and 6 months after RT. Generalized estimating equations (GEE) were used to analyze functional capacity and flexibility, while the cumulative incidence of new depressive symptoms was estimated among participants without depressive symptoms at baseline.

**Results:**

No significant group × time interactions were observed for handgrip strength (Wald χ^2^ = 7.834; df = 4; *p* = 0.098), the 2-Minute Step Test (Wald χ^2^ = 6.877; df = 4; *p* = 0.143), or flexibility (Wald χ^2^ = 3.728; df = 4; *p* = 0.444). During the RT period, the cumulative of new depressive symptoms was 14.8%.

**Conclusion:**

Functional capacity and flexibility improved over time, but their trajectories did not differ significantly between the Mat Pilates and control groups. No significant between-group difference was observed in the cumulative incidence of new depressive symptoms.

**Supplementary Information:**

The online version contains supplementary material available at https://doi.org/10.1007/s00520-026-11448-6.

## Introduction

Breast cancer remains the most common cancer among women globally, with approximately 2.3 million new cases predicted for 2022 [1]. Radiation therapy (RT) is a standard treatment following breast-conserving surgery or mastectomy [2], effectively reducing the risk of local recurrence in invasive and non-invasive breast cancer by 60% to 70% and 50% to 60%, respectively [3]. A recent trial with over 30 years of follow-up showed that RT significantly reduced the risk of local recurrence in women with early breast cancer [4].

Nevertheless, adjuvant RT can lead to both short-term (e.g., fatigue, skin irritation) [5] and long-term side effects (e.g., lymphedema) [6], along with psychological effects such as anxiety and depression [7], which can adversely affect health-related quality of life (HRQoL) [8].

Physical inactivity among women with breast cancer undergoing adjuvant treatment may interfere with self-care, daily activities, and return to work, resulting in a decline in physical functioning [9]. Sedentary time (ST) has been observed to be associated with pain four years after treatment among breast cancer survivors, while depressive symptoms are associated with elevated ST levels [10].

Engaging in physical exercise according to the American College of Sports Medicine (ACSM) guidelines can mitigate or even prevent the adverse effects associated with a cancer diagnosis and its treatment [11]. A widely studied effect of physical exercise in women with breast cancer during radiotherapy is fatigue. Torresʼs (2022) meta-analysis indicated that combining resistance training with aerobic training is the most effective type of physical exercise in reducing fatigue [12], and Zhengʼs (2025) meta-analysis also indicated a significant reduction in fatigue when performing various types of exercise (such as yoga, running, Qi Gong, resistance training, and aerobic exercise), while no significant effect was observed on depression [13]. Regarding physical performance, another recent meta-analysis by Trommer (2023) indicated positive values, but very low certainty regarding the effect of physical exercise during adjuvant radiotherapy in breast cancer [14].

The Pilates method, developed by Joseph Pilates in the 1920 s [15], incorporates a mind–body approach through exercises combining light to moderate physical activity with mindfulness [16] and it is considered a safe exercise modality for women with breast cancer [17]. Several trials have reported improvements in functional capacity [18], strength and flexibility [19], joint pain [20], upper-limb function [21], range of motion (ROM) [22], lymphedema, and anxiety [23].

However, the number of clinical trials documenting the beneficial effects of Pilates in breast cancer treatment is still low [17] and limited to studies conducted after adjuvant treatments. In fact, there are currently no clinical trials of Pilates in this population during adjuvant radiotherapy, a common treatment for breast cancer. In this context, our study fills this gap and aimed to explore the effects of Pilates Mat on functional capacity, flexibility, and depressive symptoms in women with breast cancer undergoing adjuvant radiotherapy.

## Methods

This clinical trial was conducted at the Cancer Hospital III of the National Cancer Institute (HCIII/INCA) between May 2017 and October 2019. The present manuscript reports prespecified secondary outcomes from the same randomized clinical trial previously published with fatigue as the primary outcome [24]. The previous publication focused on fatigue, physical activity level, adherence to the Mat Pilates program, and adverse events, whereas the present study evaluates functional capacity, flexibility, and depressive symptoms in the same randomized cohort. The study was registered on ClinicalTrials.gov (NCT03333993) and received approval from the Ethics Committee in Research of INCA (CAAE: 64,099,717.7.0000.5274) and of the National Public Health School (CAAE: 64,099,717.7.3001.5240). The trial was retrospectively registered after participant recruitment had begun.

Women attending their first medical appointment at the HCIII/INCA radiotherapy outpatient clinic were screened for eligibility. Women aged 18 years or older with stage 0–IIIC breast cancer and an indication for adjuvant radiotherapy at HCIII/INCA were invited to participate. Those who agreed to participate signed the informed consent form and underwent baseline assessment and randomization. Exclusion criteria comprised prior cancer diagnoses, engaging in physical exercise at least twice a week for 40 min, inability to complete questionnaires, and any conditions preventing participation in Mat Pilates (e.g., immediate breast reconstructions, acute infections, or decompensated disorders).

**Fig. 1:**
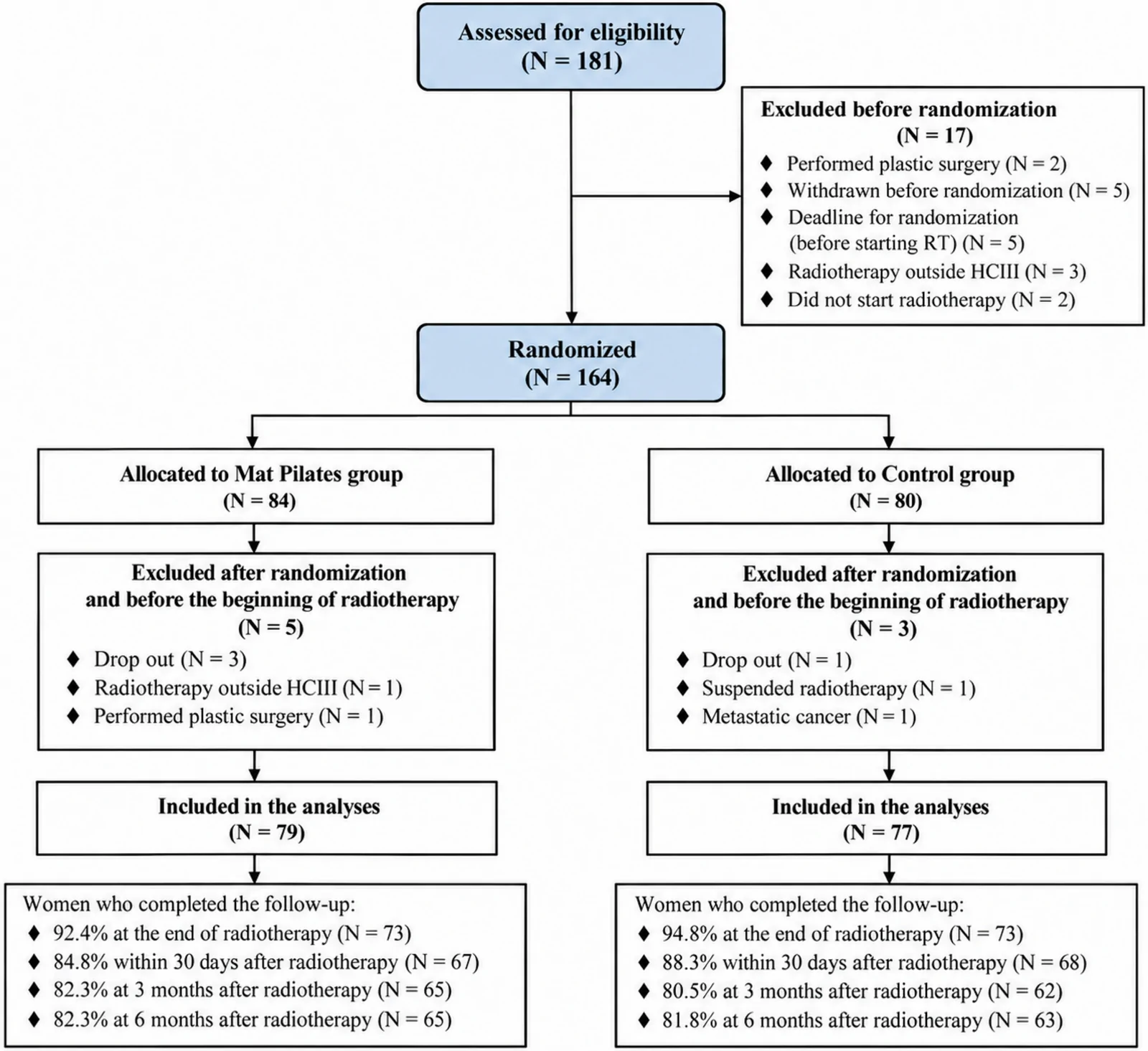
CONSORT flowchart of patients included in this study

A total of 181 women were assessed for eligibility, of whom 17 were excluded before randomization: two underwent plastic surgery, five withdrew before randomization, five could not be randomized within the required timeframe before starting RT, three underwent RT outside HCIII, and two did not start RT. Eligible participants were randomly assigned in blocks of 8 (allocation concealment was achieved using consecutively numbered, sealed, and opaque envelopes containing the allocation code) to the intervention and control groups (1:1 allocation) using a non-permuted randomization scheme prior to the start of RT. A total of 164 women were randomized, with 84 allocated to the Mat Pilates group and 80 to the control group. After randomization and before the beginning of RT and the study intervention/follow-up, eight participants were excluded: five from the Mat Pilates group and three from the control group. All eight participants had completed the baseline assessment but had no post-randomization follow-up data. Consequently, 156 participants were included in the analyses, comprising 79 women in the Mat Pilates group and 77 in the control group (Fig. 1).

It was not possible to blind participants, physical therapists supervising the exercises, or outcome assessors to group allocations.

### Intervention

Participants in the intervention group engaged in a Mat Pilates program based on Classic Pilates exercises [25]. Sessions were conducted at the HCIII/INCA physiotherapy gym from the beginning to the end of RT, comprising 7 to 12 sessions over three to six weeks. Sessions were performed twice weekly, lasted approximately 60 min, and were conducted in groups of up to four participants. The intervention was delivered by physiotherapists trained and certified in the Pilates method who received specific training in the study protocol before the intervention began to standardize its delivery.

Each session consisted of approximately 5 min of warm-up, 50 min of strengthening and flexibility exercises, and 5 min of relaxation and stretching. The specific exercises performed each week, including the equipment, sets, and repetitions, are detailed in Supplementary Table 1. Exercises were performed slowly, with the main exercises generally consisting of one set of 8–10 repetitions. The program progressed weekly, and progression was individualized according to each participantʼs performance and tolerance. Exercise difficulty or resistance was increased when the participant was comfortable at the new level and was able to perform at least eight repetitions, through strategies such as increasing exercise intensity or range of movement and reducing limb support.

Individual modifications were made when clinically necessary. Full upper-limb stretching was avoided in participants experiencing acute pain or tissue sensitivity in the irradiated region; full body-weight loading through the affected upper limb was avoided in the presence of acute heaviness or swelling; and positions that increased stress on painful joints were modified or cushioning/support was provided. Exercises were performed using mats, Swiss balls (55- and 65-cm diameter), and yellow or red elastic bands (TheraBand), corresponding to light or moderate resistance. Exercise intensity was monitored at each session using an adapted Borg Rating of Perceived Exertion Scale ranging from 0 to 10, with perceived exertion during the intervention ranging from 2 (very light) to 7 (moderate-to-intense).

To promote intervention fidelity, the weekly exercise protocol was standardized across physiotherapists, attendance was recorded at each session, and exercise intensity was monitored throughout the intervention. Participants received telephone reminders regarding scheduled sessions when necessary. Any adverse events reported during the sessions were recorded on participant evaluation forms. Exercise was discontinued in the event of cardiac decompensation, blood pressure abnormalities, or oxygen desaturation.

Both the intervention and control groups received usual care, including prescribed home upper-limb exercises from the first day after surgery and instructions to resume their usual physical activities as tolerated. Participants in the intervention group additionally received the supervised Mat Pilates program described above.

### Outcomes

Functional capacity was assessed using two physical performance tests: handgrip strength and the 2-Minute Step Test (2MST). Handgrip strength was assessed only in the dominant hand using a hand dynamometer (Kratos manual model ZM), with participants seated with the arm adducted parallel to the trunk, the shoulder in neutral rotation, and the elbow flexed at 90°. Three measurements were obtained, with a minimum interval of 30 s between attempts, and the highest value was used in the analyses. Handgrip strength was expressed in kilogram-force (kgf). The limb assessed was determined by hand dominance rather than by its relationship to the side of breast surgery; therefore, both limbs were not assessed. No formal equipment calibration procedure was performed as part of the study protocol.

Functional exercise capacity was assessed using the 2-Minute Step Test (2MST). Participants were instructed to march in place for 2 min, raising each knee to a predefined target height corresponding to the midpoint between the patella and the anterior superior iliac spine. A step was counted each time the knee reached the target height, and performance was expressed as the number of steps completed during the 2-min test.

Flexibility was assessed using the sit-and-reach test with a Wells Bench, a wooden box measuring 30.5 × 30.5 cm with a measuring scale attached to its upper surface. Participants sat with their knees extended and bare feet resting against the box and reached forward as far as possible. The maximal position was maintained for approximately 2 s while the measurement was recorded. Three attempts were performed, and the highest value achieved was recorded in centimeters (cm).

Depressive symptoms were assessed using the 15-item Geriatric Depression Scale (GDS-15), with scores ranging from 0 to 15. The Brazilian version of the GDS-15 was validated by Paradela et al. [26], who identified a cutoff of 5/6 as appropriate for screening depressive symptoms; therefore, participants with scores ≤ 5 were classified as without depressive symptoms and those with scores ≥ 6 as having depressive symptoms. Although originally developed for older adults, the GDS-15 has also demonstrated good classification accuracy in younger and middle-aged adults. Guerin et al. [27] evaluated the instrument in adults aged 18–54 years and found classification accuracy comparable to that observed in adults aged 55–80 years. Accordingly, the GDS-15 was considered suitable for screening depressive symptoms across the age range included in the present study.

### Statistical analysis

The sample size was calculated based on fatigue, the primary outcome of the original randomized clinical trial. The calculation assumed an expected between-group difference of 0.9 points and a standard deviation of 2 points, corresponding to a standardized effect size of approximately 0.45. These assumptions were defined a priori considering previous randomized trials evaluating exercise interventions and fatigue in women undergoing adjuvant treatment for breast cancer [28, 29]. A total sample of 156 participants (78 per group; 1:1 allocation) was estimated to provide 80% power at a two-sided significance level of 5%, using a two-tailed calculation based on the t statistic and non-centrality parameter. No additional allowance for potential attrition was included in the sample size calculation. The sample size was therefore determined for fatigue, the primary outcome of the original trial, and was not specifically calculated for the secondary outcomes evaluated in the present analysis.

Statistical analyses included 156 participants (79 in the Mat Pilates group and 77 in the control group). Eight participants who had been randomized (five to the Mat Pilates group and three to the control group) were not included in the analyses because they had no post-randomization follow-up data. Analyses were conducted using SPSS version 24. The generalized estimating equation (GEE) method was applied to evaluate the effects of group, time, and the group × time interaction on functional capacity and flexibility across the study assessments (baseline, end of RT, 30 days post-RT, 3 months post-RT, and 6 months post-RT). Estimates are presented with 95% confidence intervals (95% CIs). A gamma distribution with an identity link function and an independent working correlation matrix was used. Post-hoc pairwise contrasts based on estimated marginal means were performed for group, time, and group × time effects, with adjustment for multiple comparisons using the Bonferroni method.

In each final model of the dependent variables, differences between groups were presented with estimates adjusted for age, occupation, and baseline values of handgrip strength, step count, or flexibility.

Additionally, the cumulative incidence of new depressive symptoms was estimated using the Kaplan–Meier method. Participants with depressive symptoms at baseline (*n* = 34) were excluded from this analysis, resulting in an at-risk population of 122 women. The event was defined as the first occurrence of depressive symptoms according to the GDS-15 cutoff during follow-up. Participants who did not develop depressive symptoms were censored at the last assessment at which they were known to be free of depressive symptoms. The log-rank test was used to compare the cumulative incidence curves between the Mat Pilates and control groups.

## Results

A total of 164 women were randomized, with 84 allocated to the Mat Pilates group and 80 to the control group. Eight participants were excluded after randomization and before the beginning of radiotherapy and study intervention/follow-up (five from the Mat Pilates group and three from the control group). Therefore, 156 participants were included in the analyses (79 in the Mat Pilates group and 77 in the control group; Fig. 1).

The baseline characteristics of the study are presented in Table 1. Most patients were not white (60.0%), housewives (74.3%), overweight (44.2%) and with advanced clinical stage (IIB to IIIC; 61.0%). At baseline, depressive symptoms were present in 19 of 79 participants (24.1%) in the Mat Pilates group and 15 of 77 participants (19.5%) in the control group.

**Table 1:** Demographic and clinical factors in Mat Pilates (*N* = 79) and controls (*N* = 77) groups, Rio de Janeiro, Brazil, 2017–2019

Variables	Mat Pilates (*n* = 79), *n* (%)	Controls (*n *= 77), *n* (%)	Chi-square *p*-value
Age in years			**0.726**
< 45	19 (24.1)	17 (22.1)	
45–55	26 (32.9)	22 (28.6)	
> 55	34 (43.0)	38 (49.4)	
Skin color			**0.203**
White	28 (35.4)	35 (45.5)	
Others	51 (64.6)	42 (54.5)	
Marital status			**0.876**
With partner	41 (51.9)	39 (50.6)	
No partner	38 (48.1)	38 (49.4)	
Education level			**0.238**
≥ 8 years of study	61 (77.2)	53 (68.8)	
< 8 years of study	18 (22.8)	24 (31.2)	
Occupation			**0.170**
Work outside	24 (30.4)	16 (20.8)	
Housewives	55 (69.6)	61 (79.2)	
Family income			**0.779**
> 1 minimum wage	55 (69.6)	52 (67.5)	
≤ 1 minimum wage	24 (30.4)	25 (32.5)	
Alcohol consumption			**0.495**
No	65 (82.3)	60 (77.9)	
Yes	14 (17.7)	17 (22.1)	
Smoking			**0.918**
No	56 (70.9)	54 (70.1)	
Former or current smoker	23 (29.1)	23 (29.9)	
Body mass index			**0.530**
Ideal	17 (21.5)	14 (18.2)	
Overweight	37 (46.8)	32 (41.6)	
Obesity	25 (31.6)	31 (40.3)	
Clinical staging			**0.282**
0/I/IIA	27 (34.2)	34 (44.2)	
IIB/IIIA	34 (43.0)	24 (31.2)	
IIIB/IIIC	18 (22.8)	19 (24.7)	
Molecular subtype			0.256*
Luminal A	19 (24.1)	14 (18.2)	
Luminal B	48 (60.8)	57 (74.0)	
HER2-positive	2 (2.5)	2 (2.6)	
Triple negative	10 (12.7)	4 (5.2)	
Breast surgery			**0.879**
Segmentectomy	37 (46.8)	37 (48.1)	
Mastectomy	42 (53.2)	40 (51.9)	
Axillary emptying			**0.879**
No	37 (46.8)	37 (48.1)	
Yes	42 (53.2)	40 (51.9)	
Chemotherapy			0.195*
No	14 (17.7)	10 (13.0)	
Neoadjuvant	49 (62.0)	41 (53.2)	
Adjuvant	16 (20.3)	25 (32.5)	
Neoadjuvant and adjuvant	0 (0.0)	1 (1.3)	
End of CT to RT			**0.330**
No	14 (17.7)	10 (13.0)	
1–3 months	10 (12.7)	18 (23.4)	
4–5 months	13 (16.5)	10 (13.0)	
≥ 6 months	42 (53.2)	39 (50.6)	
Radiotherapy site			0.570*
Breast/Plastron	19 (24.1)	21 (27.3)	
Breast/Plastron and SCF	50 (63.3)	42 (54.5)	
Breast/Plastron and armpit	0 (0.0)	1 (1.3)	
Breast/Plastron/SCF and armpit	10 (12.7)	13 (16.9)	
Radiotherapy mode			**0.511**
Hypofractionated	11 (13.9)	14 (18.2)	
Conventional	58 (73.4)	57 (74.0)	
Boost	10 (12.7)	6 (7.8)	
Technique of radiotherapy**			**0.560**
Two-dimensional (2D)	62 (81.6)	64 (84.2)	
Three-dimensional (3D)	14 (18.4)	12 (15.8)	
Target therapy			**0.948**
No	68 (86.1)	66 (85.7)	
Yes	11 (13.9)	11 (14.3)	
Hormone therapy			**0.267**
No	13 (16.5)	8 (10.4)	
Yes	66 (83.5)	69 (89.6)	

*CT* chemotherapy, *HER2* human epidermal growth factor receptor 2, *IDC* invasive ductal carcinoma, *RT* radiotherapy, *SCF* supraclavicular fossa. * Fisher exact test. ** Total variations due to missings in variables.

The number of prescribed Mat Pilates sessions varied from 7 to 12 according to the duration of each participantʼs RT schedule. Adherence was calculated as the percentage of individually prescribed sessions attended. Median adherence was 71.0% (IQR, 36), and 53 of 79 participants (67.1%) attended ≥ 70% of their prescribed sessions. In addition, 19 participants (24.1%) completed ≥ 90% of their prescribed sessions. Two participants discontinued Mat Pilates after four and five sessions because of surgical-site discomfort associated with radiodermatitis, two discontinued after five and six sessions because of joint discomfort, one discontinued after eight sessions because severe radiodermatitis resulted in surgical wound opening and suspension of RT, and one discontinued after five sessions because of a foot fracture that occurred outside the Mat Pilates program. None of these participants withdrew from the study because of these events.

The estimated means for handgrip strength, the 2-Minute Step Test, and flexibility across the study assessments, together with the GEE results for group and time effects, are presented in Table 2. No significant global group × time interaction was observed for handgrip strength (Wald χ^2^ = 7.834; df = 4; *p* = 0.098), the 2-Minute Step Test (Wald χ^2^ = 6.877; df = 4; *p* = 0.143), or flexibility (Wald χ^2^ = 3.728; df = 4; *p* = 0.444), indicating that the trajectories of these outcomes over time did not differ significantly between the Mat Pilates and control groups. Post-hoc between-group contrasts at each assessment point, derived from the estimated marginal means and adjusted for multiple comparisons using the Bonferroni method, are presented in Table 3.

**Table 2:** Estimated means by group, time (combined mean), standard error (SE), unadjusted and adjusted mean difference (95% confidence interval) for functional capacity and flexibility (*n* = 156)

	Time	Estimated mean by group and combined mean (SE)		Unadjusted Mean Difference						Adjusted mean Difference*					
				Between-group difference			Within-group difference (Time)			Between-group difference			Within-group difference (Time)		
		Mat Pilates	Controls	Mean Difference 95% CI		**p-value	Mean Difference 95% CI		**p-value	Mean Difference 95% CI		**p-value	Mean Difference 95% CI		**p-value
	.	22.50 (11.53)	23.40 (11.49)	0.249	(-1.19, 1.69)	0.734	.	.	.	-0.906	(-1.73,-0.08)	**0.032**	.	.	.
Handgrip strength	**Baseline**	21.84 (11.61)		.	.	.	Ref.	.	.	.	.	.	Ref.	.	.
	**End of RT**	23.16 (11.48)		.	.	.	0.627	(-0.32 1.58)	0.637	.	.	.	1.323	(0.14, 2.50)	**0.016**
	**30 days after RT**	23.14 (11.46)		.	.	.	0.444	(-0.62, 1.51)	1.000	.	.	.	1.298	(0.06, 2.54)	**0.034**
	**3 months after RT**	22.86 (11.53)		.	.	.	0.808	(-0.37, 1.99)	0.549	.	.	.	1.022	(-0.23, 2.28)	0.224
	**6 months after RT**	23.76 (11.46)		.	.	.	1.290	(-0.01, 2.59)	0.052	.	.	.	1.918	(0.55, 3.29)	**0.001**
	.	201.76 (450.28)	199.97 (449.21)	6.130	(-8.35, 20.61)	0.407	.	.	.	1.790	(-6.35, 9.93)	0.666	.	.	.
Number of steps taken	**Baseline**	191.56 (449.58)		.	.	.	Ref.	.	.	.	.	.	Ref.	.	.
	**End of RT**	198.82 (449.96)		.	.	.	5.000	(-5.14, 15.11)	1.000	.	.	.	7.260	(-2.15, 16.67)	0.304
	**30 days after RT**	203.92 (449.83)		.	.	.	11.760	(1.64, 21.89)	**0.011**	.	.	.	12.350	(3.45, 21.25)	**0.001**
	**3 months after RT**	207.77 (449.29)		.	.	.	15.660	(4.01, 27.31)	**0.002**	.	.	.	16.200	(5.71, 26.70)	**<0.001**
	**6 months after RT**	202.27 (450.08)		.	.	.	9.280	(-2.20, 20.76)	0.233	.	.	.	10.710	(1.04, 20.37)	**0.019**
	.	21.23 (14.14)	20.21 (14.23)	1.027	(-1.35, 3.40)	0.397	.	.	.	1.019	(-0.07, 2.10)	0.065	.	.	.
Flexibility	**Baseline**	19.44 (14.24)		.	.	.	Ref.	.	.	.	.	.	Ref.	.	.
	**End of RT**	21.13 (14.17)		.	.	.	1.205	(0.03, 2.38)	**0.039**	.	.	.	1.685	(0.54, 2.83)	**<0.001**
	**30 days after RT**	21.07 (14.22)		.	.	.	1.331	(-0.06, 2.72)	0.073	.	.	.	1.631	(0.29, 2.98)	**0.007**
	**3 months after RT**	21.38 (14.17)		.	.	.	1.278	(-0.20, 2.75)	0.149	.	.	.	1.940	(0.27, 3.62)	**0.011**
	**6 months after RT**	20.58 (14.12)		.	.	.	0.928	(-0.86, 2.71)	1.000	.	.	.	1.143	(-0.24, 2.53)	0.207

**Table 3:** Estimated means by Group * Time, standard error (SE), unadjusted and adjusted mean difference (95% confidence interval) for functional capacity and flexibility (*n* = 156)

	Time	Mean (SE)		Unadjusted Mean Difference			Adjusted Mean Difference *		
				Group * Time			Group * Time		
		Mat Pilates	Controls	Mean Difference 95% CI		**p-value	Mean	Difference 95% CI	**p-value
Handgrip strength	**Baseline**	22.11 (11.57)	21.57 (11.66)	Ref			Ref		
	**End of RT**	22.53 (11.52)	23.79 (11.45)	0.010	(− 2.57, 2.59)	1.000	−1.261	(− 3.47, 0.95)	1.000
	**30 days after RT**	22.17 (11.54)	24.11 (11.39)	−0.621	(− 3.44, 2.20)	1.000	−1.941	(− 4.38, 0.50)	0.426
	**3 months after RT**	22.53 (11.55)	23.20 (11.53)	−0.013	(− 3.31, 3.29)	1.000	−0.670	(− 3.42, 2.08)	1.000
	**6 months after RT**	23.15 (11.48)	24.36 (11.46)	0.509	(− 2.85, 3.87)	1.000	−1.205	(− 3.91, 1.50)	1.000
Number of steps taken	**Baseline**	191.00 (449.69)	192.12 (449.48)	Ref			Ref		
	**End of RT**	204.34 (450.53)	193.31 (449.41)	15.830	(− 13.00, 44.65)	1.000	11.030	(− 10.16, 32.21)	1.000
	**30 days after RT**	205.12 (450.14)	202.71 (449.53)	3.690	(− 25.65, 33.04)	1.000	2.410	(− 17.60, 22.42)	1.000
	**3 months after RT**	209.00 (450.02)	206.53 (448.60)	10.860	(− 20.39, 42.11)	1.000	2.470	(− 20.80, 25.75)	1.000
	**6 months after RT**	199.36 (451.07)	205.19 (449.12)	0.160	(− 32.23, 32.55)	1.000	−5.840	(− 27.81, 16.14)	1.000
Flexibility	**Baseline**	19.53 (14.25)	19.35 (14.23)	Ref			Ref		
	**End of RT**	21.59 (14.11)	20.67 (14.25)	1.279	(− 3.19, 5.74)	1.000	0.922	(− 1.63, 3.48)	1.000
	**30 days after RT**	21.99 (14.13)	20.15 (14.32)	0.679	(− 3.87, 5.22)	1.000	1.838	(− 1.12, 4.80)	1.000
	**3 months after RT**	21.74 (14.04)	21.02 (14.33)	2.131	(− 2.56, 6.82)	1.000	0.715	(− 2.99, 4.42)	1.000
	**6 months after RT**	21.30 (14.18)	19.86 (14.08)	1.045	(− 3.83, 5.92)	1.000	1.439	(− 1.84, 4.71)	1.000
*Adjusted for baseline value of the outcomes, age, and occupation **Multiple comparisons of means were adjusted using the Bonferroni method									

### Functional capacity

The results of the GEE analyses for handgrip strength and the 2-Minute Step Test are presented in Tables 2 and 3.

In the adjusted GEE analysis, the overall mean handgrip strength was lower in the Mat Pilates group than in the control group (MD: − 0.91 kgf; 95% CI: − 1.73 to − 0.08). However, no significant group × time interaction was observed for handgrip strength in either the unadjusted (Wald χ^2^ = 7.735; df = 4; *p* = 0.102) or adjusted model (Wald χ^2^ = 7.834; df = 4; *p* = 0.098), indicating that changes in handgrip strength over time did not differ significantly between the groups.

For the 2-Minute Step Test, the overall adjusted mean difference between the Mat Pilates and control groups was 1.79 steps (95% CI: − 6.35 to 9.93). No significant group × time interaction was observed in either the unadjusted (Wald χ^2^ = 7.220; df = 4; *p* = 0.125) or adjusted model (Wald χ^2^ = 6.877; df = 4; *p* = 0.143), indicating that changes in functional exercise capacity over time did not differ significantly between the groups.

Considering both groups together, the adjusted estimated marginal means showed significant increases in handgrip strength from baseline to the end of RT (MD: 1.32 kgf; 95% CI: 0.14 to 2.50), 30 days (MD: 1.30 kgf; 95% CI: 0.06 to 2.54), and 6 months after RT (MD: 1.92 kgf; 95% CI: 0.55 to 3.29). The number of steps completed during the 2MST was also significantly higher than baseline at 30 days (MD: 12.35 steps; 95% CI: 3.45 to 21.25), 3 months (MD: 16.20 steps; 95% CI: 5.71 to 26.70), and 6 months after RT (MD: 10.71 steps; 95% CI: 1.04 to 20.37).

### Flexibility

The results of the GEE analyses for flexibility are presented in Tables 2 and 3. In the adjusted GEE analysis, the overall mean difference in flexibility between the Mat Pilates and control groups was 1.02 cm (95% CI: − 0.07 to 2.10). No significant group × time interaction was observed in either the unadjusted (Wald χ^2^ = 5.405; df = 4; *p* = 0.248) or adjusted model (Wald χ^2^ = 3.728; df = 4; *p* = 0.444), indicating that changes in flexibility over time did not differ significantly between the groups.

Considering both groups together, flexibility increased significantly from baseline to the end of RT (MD: 1.69 cm; 95% CI: 0.54 to 2.83), 30 days after RT (MD: 1.63 cm; 95% CI: 0.29 to 2.98), and 3 months after RT (MD: 1.94 cm; 95% CI: 0.27 to 3.62).

### Depressive symptoms

Participants with depressive symptoms at baseline were excluded from the survival analysis. Therefore, this analysis included 122 participants without depressive symptoms at baseline and evaluated the occurrence of new depressive symptoms during follow-up. The cumulative incidence of new depressive symptoms was 14.8% at the end of RT, 23.9% at 30 days, 25.8% at 3 months, and 26.8% at 6 months after RT. Only one new case occurred between the 3- and 6-month assessments. There was no statistically significant difference in cumulative incidence between the Mat Pilates and control groups (log-rank *p* = 0.909). When the follow-up intervals were considered separately, the largest number of new cases occurred by the end of RT (Table 4).

**Table 4:** Cumulative incidence of new depressive symptoms among participants without depressive symptoms at baseline, overall and according to study group (*n* = 122)

Time interval	Number at risk	New cases of depressive symptoms	Cumulative incidence of depressive symptoms (%)	95% CI of cumulative incidence
Total				
End of RT	122	18	14.8	8.2, 20.8
30 days after RT	102	11	23.9	15.9, 31.2
3 months after RT	83	2	25.8	17.5, 33.2
6 months after RT	71	1	26.8	18.4, 34.4
Mat Pilates Group				
End of RT	60	9	15.0	5.5, 23.6
30 days after RT	50	6	25.2	13.3, 35.5
3 months after RT	41	1	27.0	14.8, 37.5
6 months after RT	33	0	27.0	14.8, 37.5
Control Group				
End of RT	62	9	14.5	5.3, 22.8
30 days after RT	52	5	22.7	11.5, 32.5
3 months after RT	42	1	24.6	12.9, 34.7
6 months after RT	38	1	26.6	14.4, 37.0

Difference in incidences between Mat Pilates and the control groups by Log-rank test: *p* = 0.909

## Discussion

In this randomized clinical trial, no significant group × time interactions were observed for handgrip strength, functional exercise capacity assessed by the 2-Minute Step Test, or flexibility, indicating that the trajectories of these outcomes did not differ significantly between the Mat Pilates and control groups. Nevertheless, significant improvements over time were observed in functional capacity and flexibility when considering the study population as a whole. These findings suggest that the improvements observed during follow-up cannot be specifically attributed to the Mat Pilates intervention. Among participants without depressive symptoms at baseline, the cumulative incidence of new depressive symptoms reached 26.8% by 6 months after RT, with no significant difference between the Mat Pilates and control groups.

Pilates is widely used in the general population, both in the clinical area and in the fitness area [30]. There is already a vast literature in healthy elderly women, as well as in middle-aged women on the gain of flexibility [31–33], functionality [34, 35], muscle strength [32, 33, 35, 36], postural balance [32–35, 37] and pain [38].

In women with breast cancer, the effects of Pilates have been investigated in previous clinical trials. Eyigor et al. [18] reported a significant difference in functional capacity between women who performed Mat Pilates three times per week and those in the control group. While functional capacity increased in the Pilates group, a reduction was observed in the control group [18]. In the present study, however, no significant group × time interaction was observed for either handgrip strength or the 2-Minute Step Test, indicating that changes in functional capacity over time did not differ significantly between the Mat Pilates and control groups. One possible explanation for the different findings may be the lower frequency of the intervention in our study (two sessions per week) compared with that used by Eyigor et al. [18]. Nevertheless, the influence of session frequency was not specifically evaluated in the present study.

In the trial by Eyigor et al. [18], flexibility was assessed using the same test employed in the present study, and no significant within- or between-group changes were observed. Barbosa et al. [20] evaluated 20 women undergoing adjuvant hormone therapy for breast cancer who performed Mat Pilates twice weekly for 8 weeks. Although flexibility improved within the Pilates group, no significant between-group difference was observed [20]. Similarly, in the present study, flexibility improved over time, but the trajectory of change did not differ significantly between the Mat Pilates and control groups.

Pilates exercises incorporate components that may contribute to postural stability, joint mobility, muscle strength, and flexibility [39]. Dynamic stretching is also incorporated into Pilates exercises and may influence flexibility [31]. However, although flexibility improved over time in the present study, the absence of a significant group × time interaction indicates that this improvement cannot be specifically attributed to the Mat Pilates intervention. The twice-weekly frequency and relatively short duration of the intervention may have limited the ability to detect additional effects of Mat Pilates, although these hypotheses were not specifically tested.

The improvement in functional capacity and flexibility observed over time in both groups may potentially be related to the increase in physical activity levels reported in our previous publication [24]. In addition, continuous participation in study assessments and encouragement to maintain usual physical activities may have contributed to these findings. However, the specific influence of these factors on functional capacity and flexibility was not directly assessed in the present study and should therefore be considered a possible explanation rather than an established mechanism.

Upper-limb muscle strength assessed by handgrip dynamometry has also been evaluated in previous clinical trials of Mat Pilates among women with breast cancer. Two trials, each including approximately 30 women per group, reported no significant between-group differences in handgrip strength after Mat Pilates performed three times per week for 8 weeks [21, 23]. Similar to our exercise program, both interventions incorporated elastic bands for muscle strengthening. In the present study, handgrip strength changed over time, but no significant group × time interaction was observed, indicating that its trajectory did not differ between the Mat Pilates and control groups. Although gains in muscle strength depend on an adequate training stimulus [39], the influence of exercise intensity was not specifically evaluated in the present study. Evidence regarding the effects of Pilates on upper-limb muscle strength also remains limited [35].

In the present study, the analysis of depressive symptoms focused on the development of new symptoms among participants without depressive symptoms at baseline. No significant difference in the incidence of new depressive symptoms was observed between the Mat Pilates and control groups during follow-up. Boing et al. [40] also found no significant effect of a 16-week Pilates intervention on depressive symptoms among breast cancer survivors. Depressive symptoms have been reported among women with breast cancer undergoing RT. In a cohort of women with a mean age of 58.6 years (SD: 12.1), 48.1% (*n* = 310) presented depressive symptoms at the end of RT [41]. In contrast, the multicenter START trial, which evaluated 1,914 women with a mean age of 56.9 years (SD: 10.4), reported a prevalence of depressive symptoms of 4.0% (*n* = 81) at 6 months after adjuvant RT [42]. Differences in the prevalence of depressive symptoms across studies may reflect variations in the populations evaluated, assessment instruments, cutoff criteria, and timing of assessment.

This study has several limitations. First, exposure to the Mat Pilates intervention was relatively short and varied according to the duration of RT, ranging from 7 to 12 sessions over three to six weeks. Adherence was moderate, with 67.1% of participants attending at least 70% of their individually prescribed sessions, which may have attenuated the ability to detect an intervention effect. Neither participants nor outcome assessors were blinded to group allocation. In addition, both groups received prescribed home upper-limb exercises and recommendations to resume their usual physical activities as tolerated; therefore, the control condition was not passive, and changes in physical activity during follow-up may have contributed to improvements over time, although this was not specifically evaluated in the present analysis. Loss to follow-up increased over time, reaching 18.6% at the 6-month assessment, which may have introduced attrition bias. In addition, eight participants were excluded after randomization because they had no post-randomization follow-up data; therefore, the analysis did not follow a conventional intention-to-treat approach. Although radiotherapy characteristics and concomitant treatments were similarly distributed between groups at baseline, heterogeneity in treatment exposure may have contributed to variability in the outcomes. The GDS-15 was used across a broad adult age range and, although evidence supports its use beyond older populations, it remains a screening instrument and does not establish a clinical diagnosis of depression. Furthermore, the sample size was calculated for fatigue, the primary outcome of the original trial, and the study may therefore have had limited statistical power to detect differences in the secondary outcomes evaluated here. Finally, the trial was registered retrospectively, after participant recruitment had begun, which should be considered when interpreting the prespecification and reporting of study outcomes.

Although no significant between-group effects were observed for functional capacity, flexibility, or the incidence of depressive symptoms in the present study, these findings do not preclude a potential role for Pilates in breast cancer rehabilitation. Upper-limb dysfunction remains an important consequence of breast cancer treatment. Moreover, a previous meta-analysis of three studies reported a favorable effect of Pilates on upper-limb function after adjuvant breast cancer treatment (summary effect: 0.94; 95% CI: 0.20 to 1.69), while a meta-analysis of two studies showed a reduction in pain (summary effect: − 0.48; 95% CI: − 0.88 to − 0.07) [17]. These findings suggest that potential benefits of Pilates may involve outcomes other than those evaluated in the present trial and warrant further investigation using adequately powered interventions with standardized exercise exposure.

## Conclusion

Mat Pilates did not result in significantly different trajectories of functional capacity or flexibility compared with the control group over the 6-month follow-up. Improvements in functional capacity and flexibility were observed over time overall, irrespective of group. The incidence of new depressive symptoms did not differ significantly between the Mat Pilates and control groups.

## Supplementary Information

Below is the link to the electronic supplementary material.

(DOCX 14.0 KB)

## Data Availability

No datasets were generated or analysed during the current study.
