# Supplementary material for: Changes in functional capacity, flexibility and depressive symptoms in women with breast cancer submitted to adjuvant radiotherapy: randomized controlled clinical trial with mat pilates
Source: Support Care Cancer. 2026 Nov 14;34(12):1224. doi: 10.1007/s00520-026-11448-6 (PMC13769710; doi:10.1007/s00520-026-11448-6)
Supplement: Supplementary file 1 [file 520_2026_11448_MOESM1_ESM.docx]

Supplementary Table 1. Weekly Mat Pilates exercise program during radiotherapy

| **Week** | **Session component** | **Exercises** | **Sets and repetitions** |
| --- | --- | --- | --- |
| **Week 1** | Warm-up | Breathing and posture awareness; Bridge | 1 set × 3 repetitions each |
|  | Exercises without equipment | The Hundred; The Roll Up; The One Leg Circle; The One Leg Stretch | 1 set × 8–10 repetitions each |
|  | Swiss ball | Little Abdominal Curls; Single Leg Stretch; On the Side; Side Stretches | 1 set × 8–10 repetitions each |
|  | Elastic bands | Kabat diagonal abduction; Triceps; Upper-limb flexion and extension; Frog Leg | 1 set × 8–10 repetitions each |
|  | Stretching | The Spine Stretch; Swiss ball: Frog Leg; Hip lateral movement and deep breathing | 1 set × 3 repetitions each |
| **Week 2** | Warm-up | Round Back | 1 set × 3 repetitions |
|  | Exercises without equipment | The Hundred (lower limbs flexed); The Side Kicking; The Saw; The Leg Pull–Front | 1 set × 8–10 repetitions each |
|  | Swiss ball | Double Leg Stretch; Hip Rolls; On the Side; Opposition of Hands and Feet | 1 set × 8–10 repetitions each |
|  | Elastic bands | Single Leg Circle; Doggie Kick; Upper-limb abduction; Rhomboid strengthening | 1 set × 8–10 repetitions each |
|  | Stretching | The Shell; Tower | 1 set × 3 repetitions each |
| **Week 3** | Warm-up | Stacking the Spine; The Spine Twist; Cat | 1 set × 3 repetitions each |
|  | Exercises without equipment | Unilateral Bridge; The Roll-Over with Legs Spread; The Leg Pull–Front; The Side Kick Kneeling | 1 set × 8–10 repetitions each |
|  | Swiss ball | The Hundred; Roll Up–Half Roll Up; Plank; Push-Up | 1 set × 8–10 repetitions each |
|  | Elastic bands | Roll Down; Rowing Front; Sitting Spiral; Upper-limb abduction | 1 set × 8–10 repetitions each |
|  | Stretching | Elastic bands: Posterior Chain and Mermaid; The One Leg Kick | 1 set × 3 repetitions each |
| **Week 4** | Warm-up | Stacking the Spine; The Spine Twist; Balance Point | 1 set × 3 repetitions each |
|  | Exercises without equipment | The Hundred (with lower-limb elevation); The Roll-Over with Legs Spread; The Corkscrew; The Side Kick Kneeling | 1 set × 8–10 repetitions each |
|  | Swiss ball | Spinal Mobilization; Hip Rolls with Balance; Plank–Single Limb Lift; Push-Up | 1 set × 8–10 repetitions each |
|  | Elastic bands | Single Leg Circle; Rowing Front; Rhomboid strengthening; Upper-limb abduction | 1 set × 8–10 repetitions each |
|  | Stretching | Elastic bands: Saw; Side Stretches | 1 set × 3 repetitions each |
| **Week 5** | Warm-up | Balance Point; Hip Up | 1 set × 3 repetitions each |
|  | Exercises without equipment | The Hundred (with lower-limb elevation); The Neck Pull; The Scissors; The Hip Twist with Stretched Arms | 1 set × 8–10 repetitions each |
|  | Swiss ball | Bend and Stretch; Roll Over; Full Roll Up; Plank–Single Limb Lift | 1 set × 8–10 repetitions each |
|  | Elastic bands | Frog Leg; Leg Pull; Rowing Round Back; Forward Trunk Flexion | 1 set × 8–10 repetitions each |
|  | Stretching | The One Leg Kick; The Shell; Swiss ball: Hip lateral movement and deep breathing | 1 set × 3 repetitions each |
| Abbreviations: RT, radiotherapy.  Notes: The Mat Pilates program was performed twice weekly during RT. Each session lasted approximately 60 minutes, including approximately 5 minutes of warm-up, 50 minutes of strengthening and flexibility exercises, and 5 minutes of relaxation and stretching. Exercises were performed slowly. The program progressed weekly, and progression was individualized according to participant performance and tolerance. Exercise difficulty or resistance was increased when the participant was comfortable at the new level and able to perform at least eight repetitions. Exercise intensity was monitored at each session using an adapted Borg Rating of Perceived Exertion Scale ranging from 0 to 10, with perceived exertion ranging from 2 (very light) to 7 (moderate-to-intense). Individual adaptations were made when clinically necessary. Swiss balls (55- and 65-cm diameter) and yellow or red elastic bands (light or moderate resistance) were used as appropriate. | | | |
